# Health-related quality of life and utility scores in short-term survivors of pediatric acute lymphoblastic leukemia

**DOI:** 10.1007/s11136-012-0183-x

**Published:** 2012-05-01

**Authors:** Raphaële R. L. van Litsenburg, Jaap Huisman, Hein Raat, Gertjan J. L. Kaspers, Reinoud J. B. J. Gemke

**Affiliations:** 1 Department of Pediatrics, VU University Medical Center Amsterdam, P.O. Box 7057, 1007 MB Amsterdam, The Netherlands; 2 Department of Medical Psychology, VU University Medical Center Amsterdam, P.O. Box 7057, 1007 MB Amsterdam, The Netherlands; 3 Department of Public Health, Erasmus University Medical Center Rotterdam, P.O. box 2040, 3000 CA Rotterdam, The Netherlands; 4 Division of Oncology-Hematology, Department of Pediatrics, VU University Medical Center Amsterdam, P.O. Box 7057, 1007 MB Amsterdam, The Netherlands

**Keywords:** Quality of life, Acute lymphoblastic leukemia, Health Utilities Index, Survivor, Childhood cancer, Pediatric

## Abstract

**Purpose:**

Increase of survival in pediatric acute lymphoblastic leukemia (ALL) has made outcomes such as health-related quality of life (HRQL) and economic burden more important. To make informed decisions on the use of healthcare resources, costs as well as utilities need to be taken into account. Among the preference-based HRQL instruments, the Health Utilities Index (HUI) is the most employed in pediatric cancer. Information on utility scores during ALL treatment and in long-term survivors is available, but utility scores in short-term survivors are lacking. This study assesses utility scores, health state, and HRQL in short-term (6 months to 4 years) ALL survivors.

**Methods:**

Cross-sectional single-center cohort study of short-term ALL survivors using HUI3 proxy assessments.

**Results:**

Thirty-three survivors (median 1.5 years off treatment) reported 14 unique health states. The majority of survivors (61 %) enjoyed a perfect health, but 21 % had three affected attributes. Overall, HRQL was nonsignificantly lower compared to the norm, although the difference was large and may be clinically relevant. Cognition was significantly impaired (*p* = 0.03).

**Conclusion:**

Although 61 % of short-term survivors of ALL report no impairment, the health status of the other patients lead to a clinically important impaired HRQL compared to norms. Prospective studies assessing utility scores associated with pediatric ALL should be performed, enabling valid and reliable cost-utility analyses for policy makers to make informed decisions.

## Introduction

Acute lymphoblastic leukemia (ALL) is the most common type of childhood cancer. Over the past decades, survival improved substantially and is now 80–85 % [1, 2]. In addition to survival and morbidity, health-related quality of life (HRQL) and cost-effectiveness of interventions are increasingly recognized as important outcome measures. In order to make informed decisions on the use of healthcare resources, the costs of interventions as well as the associated utilities need to be taken into account. Utility scores are derived from preference-based HRQL measures and can be used for the calculation of quality-adjusted life years (QALY). QALY are valuable in economic evaluations because they incorporate the gained life years as well as the quality of the life years, and thus allow for more deliberated decision making.

Among the available preference-based HRQL instruments in pediatric oncology, the Health Utilities Index (HUI) is frequently employed [3]. Most studies that have used the HUI in pediatric ALL involved either long-term survivors (>4 years) [4–6] or children on active treatment [7–9]. Information on utility scores and HRQL measured with the HUI in the years in between (i.e., in short-term survivors) is, however, still lacking but is essential to perform robust cost-utility analysis.

The aim of the present study was twofold: (1) to present utility scores and (2) to assess health state and HRQL, in short-term survivors of pediatric ALL.

## Methods

### Patients

A single-center cohort of parents of ALL survivors (≥5 years of age) was invited to participate in a cross-sectional HRQL assessment. Survivors were 6 months to 4 years after the end of treatment with no signs of recurrence. Parents were required to be fluent in Dutch. A sample size of 27 was necessary to detect significant differences between ALL patients and norms [5] with 80 % power and an effect size of 0.80 at a 5 % significance level (two-sided test). The study, involving the participation of healthy adults as proxy respondents, was waived submission for full consideration by the review board of our institution. All participating parents gave their informed consent.

### Instrument

The 15-question parent-proxy format of the Health Utilities Index Mark 3 (HUI3) was used [3]. It consists of eight attributes (vision, hearing, speech, ambulation, dexterity, cognition, pain, and emotion), which can be described in 5 or 6 levels and describe the patient’s health state. Level 1 represents no impairment, and higher levels represent more severe impairment. Attribute levels are used to determine single-attribute utility (SAU) scores and multi-attribute utility (MAU) scores using published utility functions. Attribute scores are regarded to represent HRQL, and MAU scores were considered to indicate overall HRQL. Scores of 0.00 represent being dead and 1.00 living in perfect health. Differences in means greater than 0.03 for MAU scores and greater than 0.05 for SAU scores between the ALL cohort and the healthy population can be considered clinically important [3]. Charts were reviewed for those children that did not participate, in order to identify impaired health states. HRQL was compared to Dutch parent-proxy norms [10, 11].

The HUI was distributed during an outpatient clinic visit or sent to the patient’s home address with a stamped return envelop. A second questionnaire was sent to the patient’s home address if it was not returned after 2–4 weeks. If the second questionnaire was not returned either, the family was regarded as not interested in participating.

### Statistical analysis

The Statistical Package for Social Sciences (SPSS) for Macintosh version 18.0 was used for data analyses. The differences in descriptive variables between participants and non-participants were calculated using Fisher’s exact test and Mann–Whitney *U* tests. Since the attribute scores were not normally distributed, Mann–Whitney *U* tests were used to assess the difference in scores between ALL patients and the norm. The effect of time off treatment, age at diagnosis, and age at survey on HRQL was assessed using Spearman’s correlation. Significance level was set at *p* < 0.05 (two-sided) for all analyses.

## Results

### Demographic variables

**Fig. 1:**
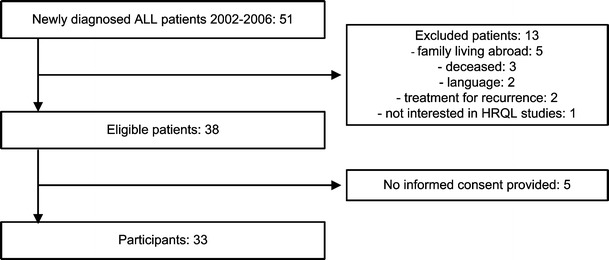
Study population: participants and non-participants

Thirty-three parents of ALL patients participated, Fig. 1. There were no missing items on the questionnaires. Median time off treatment was 1.5 years (range 0.5–3.9). None of the children were irradiated or received a stem cell transplantation. There were no differences in age or gender between the participants and the non-participants, Table 1. Chart review of the non-participants did not reveal any health state impairments.

**Table 1:** Demographic variables of the ALL patients

	Participants	Non-participants	*p*
*N* ^a^	33	18	–
Boys (%)	66 %	53 %	0.39
Age at diagnosis (years, mean ± SD)	5.5 ± 3.2	5.5 ± 3.5	0.95
Age at study (years, mean ± SD)	9.3 ± 3.3	NA	–
Median years since end off treatment (range)	1.5 (0.5–3.9)	NA	–

^a^One questionnaire was returned without identification. The demographic variables of this unknown patient were therefore analyzed in the non-participant group. *NA* not applicable

### Health-related quality of life

A total of 14 unique health states were found, Table 2. The majority of children (*n* = 20, 61 %) enjoyed a perfect health state. Impairments on three or more attributes were reported for seven (21 %) children. Over 90 % of participants had no impairment on the attributes vision, ambulation, hearing, and dexterity, Table 3. Impairment was most often reported for cognition, and it was the only attribute on which level four (“somewhat forgetful, and have a little difficulty when trying to think or solve day-to-day problems”) occurred (*n* = 3, 9 %).

**Table 2:** Frequency distribution of unique HUI3 health state vectors in the ALL population

Attribute	Number of affected attributes													
	0	1	1	1	2	2	2	3	3	3	3	3	3	4
Vision	1	2	1	1	2	1	1	1	1	2	1	1	1	1
Hearing	1	1	1	1	1	1	1	1	1	1	1	1	1	1
Speech	1	1	1	1	1	2	1	1	1	1	3	3	3	2
Ambulation	1	1	1	1	1	1	1	3	1	1	1	1	1	1
Dexterity	1	1	1	1	1	1	1	2	2	1	1	1	1	1
Emotion	1	1	1	2	2	1	1	1	1	1	1	2	1	2
Cognition	1	1	2	1	1	2	2	1	3	3	2	4	4	4
Pain	1	1	1	1	1	1	3	2	2	2	2	1	2	2
Total patients (*N*, %)	20 (61 %)	1 (3 %)	1 (3 %)	1 (3 %)	1 (3 %)	1 (3 %)	1 (3 %)	1 (3 %)	1 (3 %)	1 (3 %)	1 (3 %)	1 (3 %)	1 (3 %)	1 (3 %)

More affected attributes indicate more impairments. Maximal possible number of affected attributes is eight

**Table 3:** Frequency distribution of HUI3 attribute levels in the ALL population

Attribute	Levels (*N*, %)					
	1	2	3	4	5	6
Vision	30 (91 %)	3 (9 %)	0	0	0	0
Hearing	33 (100 %)	0	0	0	0	0
Speech	28 (85 %)	2 (6 %)	3 (9 %)	0	0	NA
Ambulation	32 (97 %)	0	1 (3 %)	0	0	0
Dexterity	31 (94 %)	1 (3 %)	1 (3 %)	0	0	0
Emotion	29 (88 %)	4 (12 %)	0	0	0	NA
Cognition	24 (73 %)	4 (12 %)	2 (6 %)	3 (9 %)	0	0
Pain	26 (79 %)	6 (18 %)	1 (3 %)	0	0	NA

Level 1 indicates no impairment; level 5 or 6 indicates the most severe impairment

*NA* not applicable

The mean MAU of the ALL patients was 0.83 compared to 0.93 in healthy children, but the difference was not statistically significant (*p* = 0.61), Table 4. Children with ALL had a significantly lower HRQL on the dexterity and cognition attribute (*p* < 0.001 and *p* = 0.03, respectively). There were no significant differences on the other attributes. There was a negative association between time off treatment and scores on the vision attribute (*r* = −0.42, *p* = 0.02). Time off treatment was not related to the other attributes, nor was age at diagnosis and age at survey.

**Table 4:** HUI3 Utility scores of the ALL patients compared to the reference population (mean ± SD)

Attribute	ALL	Reference population^a^	Difference^b^	*p* value
Vision	0.996 ± 0.015	0.998 ± 0.015	0.002	0.59
Hearing	1.000 ± 0.000	0.999 ± 0.014	0.001	0.46
Speech	0.959 ± 0.103	0.991 ± 0.027	0.032	0.41
Ambulation	0.990 ± 0.057	0.999 ± 0.015	0.009	0.15
Dexterity	0.993 ± 0.029	0.999 ± 0.010	0.006	<*0.001*
Emotion	0.989 ± 0.030	0.988 ± 0.037	−0.001	0.49
Cognition	0.951 ± 0.094	0.983 ± 0.052	0.032	*0.03*
Pain	0.979 ± 0.049	0.989 ± 0.034	0.010	0.49
Multi-attribute score	0.830 ± 0.266	0.929 ± 0.124	0.099	0.61

Higher scores indicate a better health-related quality of life

The values in italics were statistically significant

^a^ The reference population consists of parent-proxy scores of 1,435 children aged 4–13 years [12, 13]

^b^ The difference in mean scores between ALL patients and the reference population. Negative differences indicate higher scores in the ALL population. Differences greater than 0.05 can be considered clinically relevant for the single-attribute score and difference greater than 0.03 for the multi-attribute score

## Discussion

The primary aim of this study was to provide utility scores for short-term survivors of ALL for future reference in economic evaluations. Economic evaluations of pediatric (oncology) interventions are emerging [12–14] and will probably prove even more necessary in the future as expensive and time-consuming health care technology evolves. Information on utilities can be essential in determining which alternative is most cost-effective. This study adds to the limited available information on utility scores in pediatric ALL. Previous ALL parent-proxy MAU scores were 0.83–0.89 in children on active treatment [7] and 0.86–0.93 in long(er)-term survivors [5, 15], compared to 0.83 in our study. The MAU scores for children on active treatment were derived from the HUI2. Even though some studies have found HUI2 scores to be 0.01–0.05 points higher compared to HUI3 scores [15, 16], the results of this study suggest that HRQL in short-term survivors (MAU 0.83) did not improve much from HRQL in children on active treatment. Meeske et al. [17] also reported a low parent-proxy rated HRQL in short-term survivors of ALL. Low parent-rated HRQL can be explained by increased distress and fear of recurrence after the completion of their child’s cancer treatment [18]. Problems in parental psychosocial functioning such as depression, worries, and psychosocial distress have all been associated with a lower perception of HRQL [19–21].

The secondary aim of this study was to assess the health state and HRQL of short-term ALL survivors. Impairments on three or more attributes occurred in 21 % of patients, but 61 % of survivors did not report any impairment. MAU scores were nonsignificantly lower compared to the norm, although the difference may be clinically relevant since the difference in scores between both groups was larger than what is considered clinically important [3]. Cognitive defects have been reported after pediatric ALL treatment with chemotherapy only, and the cognition attribute was the most seriously and statistically significantly affected [22]. A statistically significantly lower HRQL in ALL patients was also found on the dexterity attribute, but with only a small difference in scores compared to the norm. This has not been described before in ALL survivors [5, 6, 16, 23] and may not be clinically relevant. Interestingly, previous research in survivors reported an impaired emotional HRQL [5, 16, 24], and it is unclear why such differences were not found in this study. Further, a significant association between impaired vision and time off treatment was found. An increased risk of cataract has been described in childhood cancer survivors treated with glucocorticosteroids [25], although no information on the cause of the impaired vision in this cohort was available.

Several limitations of this study have to be mentioned. It is a cross-sectional, single-center study with a relatively small number of patients and a skewed distribution of scores, since for most patients, no impairments were reported. This may have lead to some of the unexpected results, such as the absence of a lower emotional HRQL in ALL patients. Further, parental reports were used because most children were too young for the use of HUI3 self-reports. Children and parents do not always agree on their perception of HRQL, and differences have been found for the HUI3 as well [6, 23, 26]. Elevated levels of psychological distress in parents [27] and adaptation to the disease process in patients [28] can both affect HRQL assessment. It would therefore seem preferable to collect both patient and proxy assessments of HRQL in the future, although in pediatric oncology, the patients are often too young or too ill.

In conclusion, information on utility scores associated with pediatric ALL is scarce but vital for policy makers make informed decisions based on valid and reliable cost-utility analyses. This study provides preliminary evidence that utility scores in short-term survivors are similar to scores in children during treatment with potentially clinically important differences in overall HRQL functioning compared to healthy controls. Rigorous longitudinal studies to assess utility scores during and after treatment for pediatric ALL are necessary in order to identify the subgroups of patients with a poorer HRQL and to perform robust cost-effectiveness analysis.
